# Multifunctional hydrogel-based engineered extracellular vesicles delivery for complicated wound healing

**DOI:** 10.7150/thno.97317

**Published:** 2024-07-08

**Authors:** Zuhao Li, Jinlong Liu, Jian Song, Zhifeng Yin, Fengjin Zhou, Hao Shen, Guangchao Wang, Jiacan Su

**Affiliations:** 1 Department of Orthopedics, Xinhua Hospital Affiliated to Shanghai Jiao Tong University School of Medicine, Shanghai, China.; 2 Organoid Research Center, Institute of Translational Medicine, Shanghai University, Shanghai, China.; 3 National Center for Translational Medicine (Shanghai) SHU Branch, Shanghai University, Shanghai, China.; 4 Department of Orthopaedics, Shanghai Zhongye Hospital, Shanghai, China.; 5 Xi'an Honghui Hospital, Xi'an Orthopedic Research Institute, Shaanxi, China.

**Keywords:** extracellular vesicles, hydrogels, delivery systems, complicated wound, biomedical applications

## Abstract

The utilization of extracellular vesicles (EVs) in wound healing has been well-documented. However, the direct administration of free EVs via subcutaneous injection at wound sites may result in the rapid dissipation of bioactive components and diminished therapeutic efficacy. Functionalized hydrogels provide effective protection, as well as ensure the sustained release and bioactivity of EVs during the wound healing process, making them an ideal candidate material for delivering EVs. In this review, we introduce the mechanisms by which EVs accelerate wound healing, and then elaborate on the construction strategies for engineered EVs. Subsequently, we discuss the synthesis strategies and application of hydrogels as delivery systems for the sustained release of EVs to enhance complicated wound healing. Furthermore, in the face of complicated wounds, functionalized hydrogels with specific wound microenvironment regulation capabilities, such as antimicrobial, anti-inflammatory, and immune regulation, used for loading engineered EVs, provide potential approaches to addressing these healing challenges. Ultimately, we deliberate on potential future trajectories and outlooks, offering a fresh viewpoint on the advancement of artificial intelligence (AI)-energized materials and 3D bio-printed multifunctional hydrogel-based engineered EVs delivery dressings for biomedical applications.

## Introduction

Wound healing involves four overlapping biological events, namely, hemostasis, inflammation, proliferation, and remodeling 1, 2. Most wounds can heal properly, however, in cases of severe conditions such as burns, diabetes, infections, and others, the process of wound healing can be significantly delayed or even fail, causing a large medical burden and decreased quality of life for patients 3, 4. However, traditional debridement, infection treatment and dressing lack individualized designs for pathological wound microenvironments, making it difficult to solve pathophysiological problems in complicated wound healing, and eventually leading to wound healing obstacles. For example, traditional therapies struggle to consistently control excessive inflammation, reactive oxygen species overproduction, bacterial infections, and impaired angiogenesis within the diabetic microenvironment. Therefore, there is an urgent need for new means to address this issue.

Extracellular vehicles (EVs) are small, membranous particles are produced by almost all cell types to facilitate communication between cells 5, 6. These vesicles contain a wide array of signaling molecules derived from their parent cells, including proteins, enzymes, cytokines, nucleic acids, lipids, metabolites, and cell surface receptors 7-9. Mounting evidence suggests that in addition to transporting these cargos to recipient cells for intercellular communication, EVs may also play crucial roles in processes such as cell differentiation, proliferation, angiogenesis, oxidative stress response, and immune signaling 10-12. One notable characteristic of EVs is their natural biocompatibility, circulation stability, low toxicity and immunogenicity, making them optimal candidates for therapeutic applications in regenerative medicine. Moreover, their efficiency in delivering molecular cargos further enhances their potential as valuable tools for developing novel therapies 13-16. As the understanding of EVs continues to grow, their significance in biomedical applications is becoming increasingly apparent. In recent years, the use of EVs in the field of wound healing has garnered increasing interest. Studies have focused on the composition of EVs and their ability to reduce inflammation, regulate the extracellular matrix (ECM), and promote angiogenesis when applied to skin wounds 17. The wound healing process is a multi-tissue coordinated pathophysiological process involving multiple tissues, usually involving hemostasis, inflammation, proliferation, and remodeling 18. EVs mediate signal transduction in all stages of physiological healing of skin wounds, with platelet and monocyte-derived EVs regulating clot formation to achieve hemostasis; neutrophil-derived EVs regulating inflammation, macrophages and endothelial progenitor cell-derived EVs driving angiogenesis, and myofibroblast-derived EVs remodeling the ECM 19, 20. Overall, the use of EVs promotes skin regeneration in both diabetic and nondiabetic wounds and affects all aspects of the healing process.

However, the clinical application of EVs is hindered by various challenges, such as their reduced bioavailability and susceptibility to environmental factors. The traditional application method of EVs involves subcutaneous injection at the wound edge, which may inflict secondary harm on the wound, leading to pain and even further tissue damage. In addition, using EVs directly as wound dressings can result in rapid clearance of bioactive substances and limited efficacy 21, 22. When administered systemically via intravenous injection, EVs are quickly eliminated by the liver and spleen, leading to only approximately 1% of EVs remaining after 24 h. This limited bioavailability poses a significant obstacle to their therapeutic effectiveness 23, 24. Furthermore, local administration of EVs presents its own set of limitations, as the EVs are rapidly eliminated after being transported to the surrounding tissues and capillaries. Repeated administration of EVs may delay the natural healing process by providing a continuous stimulus to the injured tissue. In addition, the accumulation of reactive oxygen species (ROS), variations in pH value and ionic components can also impact the stability of EVs, further complicating their clinical use 25. To address the rapid clearance of EVs when administered intravenously, subcutaneously, or intraperitoneally, finding suitable biomaterials as vesicle release systems has become a major research focus for the successful translation of EVs-based therapies into clinical practice.

To address these obstacles, there has been a growing focus on the use of three-dimensional (3D) materials, with hydrogels in particular garnering attention for their potential in promoting wound healing 26, 27. Hydrogels, composed of 3D polymer networks, exhibit a high-water content, and they are designed to mimic the ECM with favorable biocompatibility and plasticity 28, 29. Hydrogel dressings are anticipated to offer a range of beneficial functions for wound care, such as offering a moist wound environment, protection from secondary infections, absorption of excessive exudate, good air permeability and so on, thus accelerating the efficiency of wound healing 30-32. Furthermore, as potential candidates for drug delivery systems, the release curves of encapsulated substances can be controlled by adjusting hydrogel properties, such as network morphology and crosslinking density, as well as hydrogel degradability 33-35. Recently, several studies have shown that the encapsulation of EVs in hydrogel wound dressings can continuously release EVs during hydrogel biodegradation, thereby improving the bioactivity and therapeutic efficiency of EVs 36, 37. Given these characteristics, hydrogels demonstrate significant promise as optimal wound dressings, because they create an environment conducive to healing and can also function as drug delivery systems, safeguarding and regulating the release of EVs.

In this review, we discuss the strategy of hydrogels as delivery systems for sustained release of EVs to promote wound healing. Furthermore, in the face of complicated wounds, functionalized hydrogels with specific wound microenvironment regulation capabilities, such as antimicrobial, anti-inflammatory, and immune regulation, have been proposed for loading engineered EVs to address these healing challenges, thus providing a novel perspective for the study and development of engineered wound dressings.

## Mechanism of EVs promoting wound healing

### Overview of EVs

**Figure 1:**
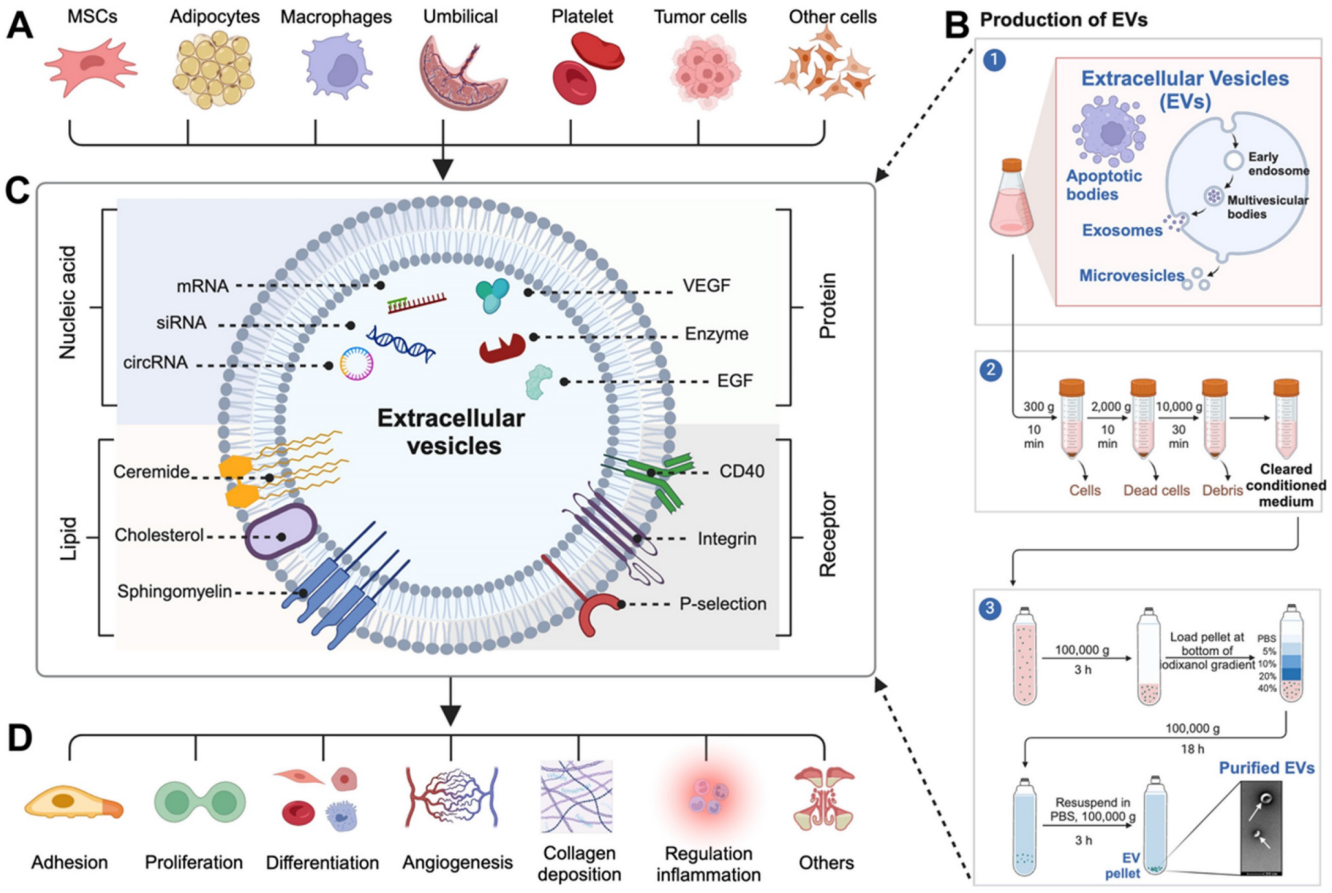
(A) EVs are derived from various cell types. (B) The production and purification process of EVs. (C) Various bioactive molecules carried on the surface and inside EVs. (D) EVs target specific cells and exert diverse biological functions.

EVs are composed of lipid bilayers and wrapped in membranous particles ranging in diameter ranging from 30 nm to 10 µm. They are produced and released by almost all cell types, such as mesenchymal stem cells (MSCs), adipocytes, platelets, macrophages, umbilical vein endothelial cells, immune cells, and etc (**Figure 1A**), and can be found in nearly all bodily fluids, including blood, saliva, urine, cerebrospinal fluid, and milk 38-40. The term EVs encompasses various types of vesicles released from cells through the well-established purification process (**Figure 1B**), including apoptotic bodies (diameter ranges from 500 nm to 10 µm), which are large vesicles released from cells undergoing apoptosis; microvesicles (diameter ranges from 200 nm to 1.0 µm), which are shed from the plasma membrane; and exosomes (diameter ranges from 30 nm to 200 nm), which are generated within multivesicular bodies and subsequently released into the extracellular fluid upon fusion of these bodies with the plasma membrane 41, 42. Initially, EVs were thought to be simply remnants of cellular debris or indicators of cell death, but further research has revealed that EVs are actually actively released by donor cells into the extracellular environment to perform a variety of important biological functions 43.

EVs are composed of the membranes and contents of their parent cells, resulting in a distinctive signature of macromolecules specific to the cell from which they originated 44-46. A wide array of signaling molecules (including proteins, enzymes, cytokines, nucleic acids, lipids, metabolites, and cell surface receptors) derived from their parent cells, are found within and on the surface of EVs (**Figure 1C**) 44, 45. When these macromolecules are released from EVs, they can trigger various responses in recipient cells, thus enabling them to perform a wide range of functions, including cell adhesion, proliferation, differentiation, angiogenesis, collagen deposition, and inflammation regulation (**Figure 1D**) 47-49. This discovery has expanded our understanding of the significance and potential applications of EVs in accelerating wound healing.

### Wound healing process and its activated mechanism by EVs

**Figure 2:**
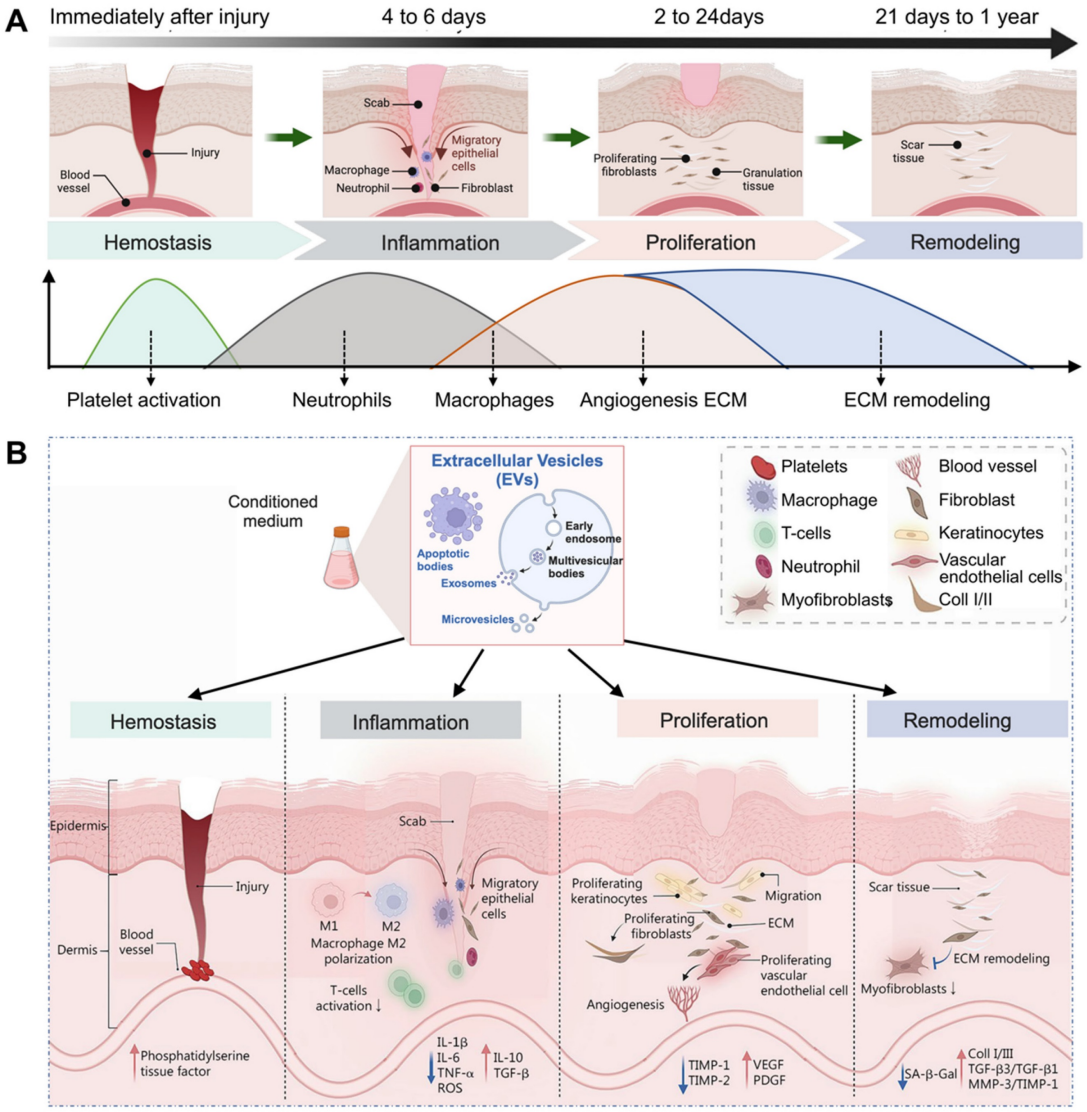
(A) Four typical biological processes of wound healing. (B) Major events in each phase of EVs-induced wound healing. Adapted with permission 53. Copyright © 2023, Springer Nature.

The process of wound repair typically involves four stages, including hemostasis, inflammation, proliferation, and remodeling (**Figure 2A**). The details are as follows 50-52. (1) Hemostasis phase: In the case of skin injury, the first stage involves blood vessel constriction and fibrin clot formation, which prevent and protect the body from blood loss. (2) Inflammatory phase: After clotting, the body releases white blood cells and other chemicals to clear out infected and dead tissue, facilitating the proliferation of wound tissue. This stage lasts 4 to 6 days and is often accompanied by redness, swelling, fever, and pain. (3) Proliferation phase: This period lasts about 2 to 24 days and can be divided into epithelial regeneration and granulation. The latter primarily involves the proliferation and differentiation of vascular endothelial cells and fibroblasts, as well as the development of new capillaries. These components work synergistically to form granulation tissue, which fills and covers wounds, ultimately leading to the formation of scars. (4) Remodeling (or maturation) phase: This period primarily involves the remodeling of scars. Following the repair process the wound achieves initial healing. Over time, the scar tissue, scabs, etc., gradually remodel to restore tissue integrity and physiological function, and eventually the appearance and function of the injured site are improved, which can generally last 21 days to 1 year.

These four intricate biological processes encompass a staggered timeline for the proliferation and differentiation of various cell types. EVs are believed to be involved in almost all processes of wound healing. The function of EVs in the wound healing process is evident in their ability to expedite the process of wound clotting; regulate the polarization of macrophages to anti-inflammatory phenotype; induce the migration, proliferation, and differentiation of skin associated cells (e.g., keratinocytes, vascular endothelial cells, and fibroblasts); remold the ECM; and exhibit anti-aging and anti-scarring effects (**Figure 2B**) 53. The specific details can be outlined as follows.

(1) Hemostasis phase: Due to the elevated levels of phosphatidylserine and tissue factor expression, EVs have a significant blood clotting effect on human blood and platelet-free plasma, thus reducing the duration of the clotting process and increasing the area of blood clots 54.

(2) Inflammatory phase: EVs are involved in promoting immunomodulatory effects, ameliorating inflammation, and producing a suitable wound healing environment. EVs have been found to have a regulatory effect on oxidative stress and inflammatory response damage induced by hyperglycemia in diabetic models 55. This regulatory effect may involve the induction of macrophage polarization to M2 phenotype and the reduction of pro-inflammatory cytokines such as interleukin (IL)-1, IL-6, tumor necrosis factor α (TNF-α), and interferon-γ (IFN-γ) 56.

(3) Proliferation phase: In this phase, EVs can induce cell proliferation and angiogenesis to promote the healing process. Specifically, in the oxidative stress microenvironment simulated by hydrogen peroxide (H_2_O_2_), EVs improved the proliferation and migration of HaCaT cells, as well as inhibited apoptosis via the miR-93-3p/APAF1 pathway 57. Previous observations indicated that EVs pretreated with DFO stimulated the PI3K/Akt signaling pathway by suppressing PTEN through the action of miR-126, which activated vascular generation *in vitro* 58. Moreover, EVs elevated the S-phase fraction of fibroblasts and promoted their proliferation capacity, ultimately contributing to the process of skin regeneration 59.

(4) Remodeling (or maturation) phase: EVs exhibit significant anti-aging and anti-scarring effects. EVs originating from adipose-derived mesenchymal stem cells (ADSCs) were able to suppress ROS accumulation and inflammatory cytokines, thus inhibiting the cellular senescence induced by high glucose levels 60. This indicates that EVs have potential applications in preventing aging-related cellular damage in diabetic individuals. Furthermore, EVs have also been found to show a critical role in remodeling the ECM by reducing the differentiation of fibroblasts into myofibroblasts through the TGF-β2/Smad2 pathway, ultimately restraining scar formation and enhancing wound healing 53, 61.

## Construction of engineered EVs

**Figure 3:**
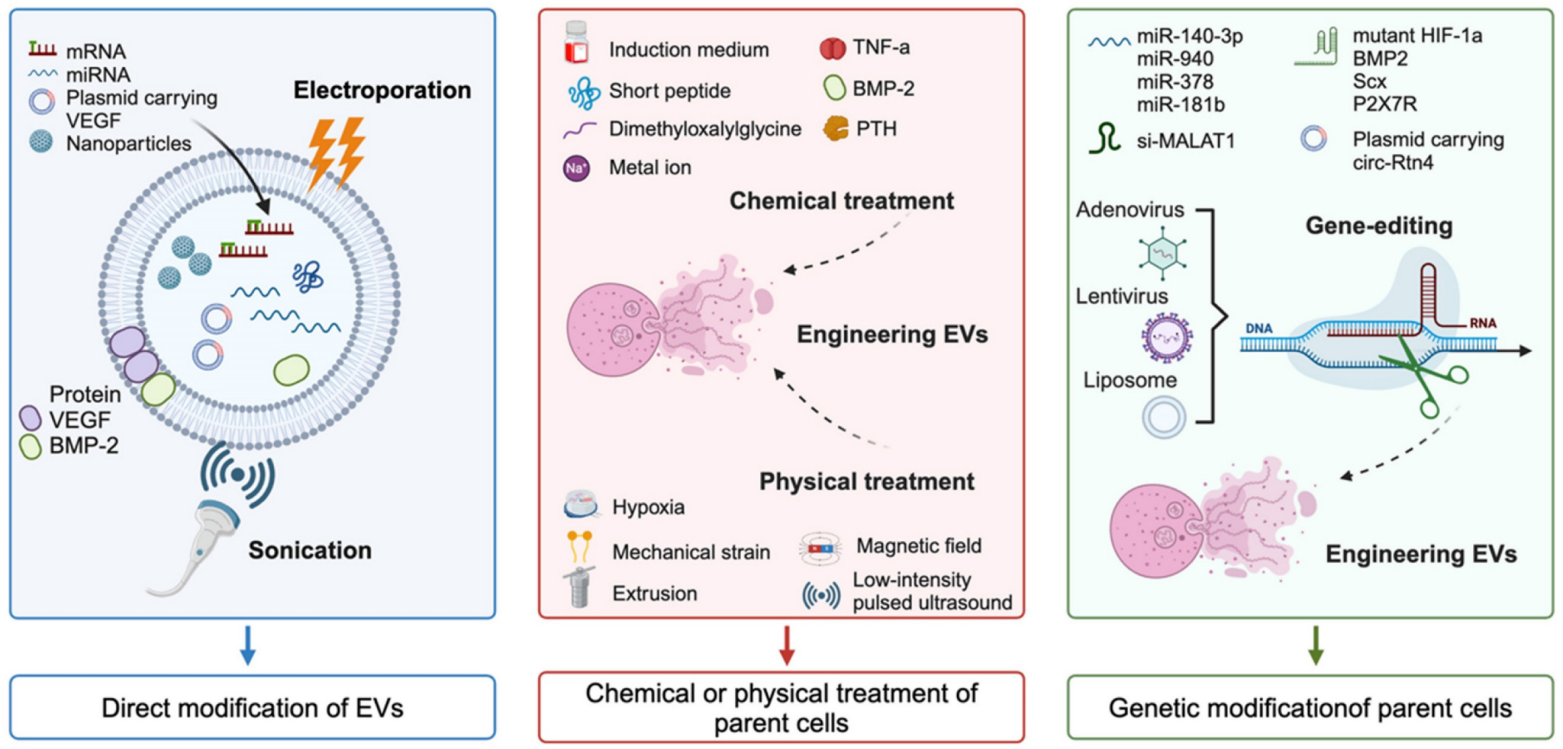
Schematic diagram illustrating the three main strategies for the construction of engineered EVs.

In the past decade, many EVs cargoes have been found to successfully promote the healing of various wounds 62, 63. However, the clinical application of EVs in wound dressings still faces significant challenges. The reasons may include low EVs yield, insufficient concentrations of bioactive cargoes, limited targeting efficiency, decreased tissue repair ability, and restricted drug delivery capabilities of native EVs 64-66. To enrich cargoes and improve the targeting efficiency of native EVs, engineered EVs have rapidly developed over the past decade, which is crucial for future clinical translation. In this chapter, we summarize three strategies for the construction of engineered EVs for tissue regeneration (**Figure 3**), namely, direct modification of EVs, chemical or physical treatment of parent cells, and genetic modification of parent cells. When constructing engineered EVs, the application scenario and mode of action should be fully considered, and the suitable construction mode for the engineered EVs should be selected. In the face of complicated application scenarios, the combination of multiple construction methods is also a strategy worth considering.

### Direct modification of EVs

Direct modification of EVs involves the enhancement of their targeting ability by decorating surface proteins, or the improvement of their regulatory function by embellishing EVs cargos or exogenous bioactive molecules through physical methods such as electroporation or sonication 67, 68, or chemical methods such as the conjugation of peptides to the surface 69. These approaches have been widely utilized to improve the targeting capacity and delivery efficiency of specific cargos to lesion regions in various diseases 70, 71. For instance, Zha *et al.* utilized an electroporation strategy to encapsulate a plasmid containing vascular endothelial growth factor (VEGF) into EVs, and these engineered EVs possessed the remarkable capability to stimulate the regeneration of vascularized tissue on a substantial scale 72. Sonication has emerged as an alternative method for loading hydrophilic molecules into EVs and has been shown to be significantly more efficient than electroporation 73. Multiple studies have shown that the combination of bone morphogenetic protein-2 (BMP-2) protein and exosomes can be sonicated to create BMP-2-loaded EVs for tissue repair 74, 75.

Direct modifications of EVs include surface modifications and internal modifications. Surface modifications can be designed to target specific cell-surface receptors on membranes, allowing for targeted delivery to specific organs, tissues, and cells. Additionally, internal modifications can be used to modify the cargo structures within EVs 76. The cargo properties can be classified into four categories: (1) small molecule drugs such as curcumin and adriamycin; (2) nucleic acids such as miRNAs, siRNAs, lncRNAs, and CRISPR/Cas9; (3) proteins; and (4) nanoparticles. Please refer to the previous review for more details 53. In summary, these modifications play a crucial role in engineering EVs for precision medicine and targeted drug delivery.

### Chemical or physical modification of parent cells

EVs, which originate from parent cells, exhibit biochemical and physiological alterations of their progenitor cells. Researches have demonstrated that pretreatment of parent cells using a variety of methods such as pharmacological agents, chemical reagents, metal ions, cytokines, hypoxia, static magnetic fields, and physical factors can enhance the function of stem cells 77-79. This finding indicates the potential for manipulating stem cells to improve the therapeutic efficacy of EVs-based treatments.

For chemical processing to prepare engineered EVs, chemical reagents and metal ions serve as two primary treatment methods. Here, growing progenitor cells in differentiation induced medium is the most commonly used strategy. For example, the engineered EVs isolated from BMSCs after osteogenic induction culture enhanced bone regeneration ability and induced rapid start of bone healing 80, 81. In addition to the induced differentiation medium, various other chemical agents, such as TNF-α 82, short peptide 83, dimethyloxalylglycine 84, and parathyroid hormone 85, have also been utilized in the production of engineered EVs for enhancing tissue repair. In addition, pretreatment with metal ions such as strontium-substituted calcium silicate ceramics and titania nanotubes in parent cells could increase the capacity of EVs to accelerate osteogenesis and angiogenesis 86, 87. In addition, a series of physical modifications of progenitor cells can also be used to prepare engineered EVs. For example, hypoxic pre-conditioning of MSCs-derived EVs promoted cartilage regeneration by the miR-205-5p/PTEN/AKT pathway 88. Parent cells acceptance of mechanically strain-derived exosomes can promote stem cell proliferation 89. To overcome the issue of low yield, an extrusion approach was used to prepare exosome mimetics from MSCs 90. In addition, magnetic nanoparticles and static magnetic fields stimulate MSCs to release miR-1260a-rich EVs, which promote osteogenesis and angiogenesis 91.

Based on the studies mentioned above, it has been established that pre-treated parent cells with chemical or physical methods is an efficient way to generate engineered EVs that can aid in tissue repair. Notably, the effectiveness of these engineered EVs is largely dependent on the cargos they carry. As such, a promising strategy for enhancing the functionality of these EVs is to modify the nucleic acid sequence of the parent cells to generate EVs with specific bioactive cargos. This alternative approach will be further discussed in the following sections.

### Genetic modification of parent cells

As molecular biology technology continues to progress, gene editing has emerged as a pivotal methodology in molecular research. By manipulating specific genes in parent cells, it is feasible to engineer EVs that contain additional or entirely novel bioactive molecules. This approach holds great promise for the creation of EVs tailored to specific therapeutic or diagnostic needs. The cargos, including miRNAs, siRNAs, lncRNAs, mRNAs, and proteins, which play a fundamental role in the function of EVs, contribute to the promotion of tissue repair and regeneration. This discovery has inspired researchers to explore the potential of creating engineered EVs through genetic modification of the parent cells.

To up-regulate the expression of miRNAs, Wang *et al.* utilized lentivirus transfection to modify BMSCs to acquire EVs overexpressing miR-140-3p, and demonstrated that this process has the capacity to induce osteogenic differentiation of MSCs and facilitate the healing of bone defects 92. In addition, mRNA editing has emerged as a crucial focal point of this strategy. For instance, Li *et al.* introduced mutated hypoxia-inducible factor-1α (HIF-1α) into BMSCs via adenovirus transfection, and the results indicated that the mutant protein was significantly expressed in BMSCs-derived EVs, which led to a substantial increase in angiogenesis and tissue regeneration 93. This finding suggests the potential for utilizing engineered EVs as a tool for promoting wound healing and regeneration through genetic modification approaches.

## Synthesis strategies of EVs combined with hydrogels

Although modification strategies have been used to address some shortcomings of EVs, they are limited by several inherent physical limitations that hinder their widespread application in nanomedicine. Similar to synthetic nanocarriers, local administration of EVs is quickly eliminated by the body or surrounding tissue upon application 94. To address the rapid clearance of locally administered EVs, finding suitable biomaterials for localized retention of vesicles and their controlled release systems has become a major research focus for the successful translation of EV-based therapies into clinical practice.

To accommodate the diverse wound microenvironments, as well as to obtain sustained release profiles of EVs and realize desired therapeutic requirements, numerous researches have been undertaken to merge EVs with hydrogels to enhance the healing process of various wounds 95. EVs release can be controlled by hydrogel properties, such as network morphology and crosslinking density, as well as hydrogel degradability 96. EVs encapsulated in the hydrogels can be continuously released during the biodegradation of hydrogels, thereby increasing the bioactivity and therapeutic efficiency of EVs 97, 98. For example, the release profiles of EVs encapsulated in a biodegradable polyethylene glycol (PEG) hydrogel can be adjusted between 6 to 27 days. Upon release from hydrogels, EVs well retain their physicochemical properties and biological functions 99.

**Figure 4:**
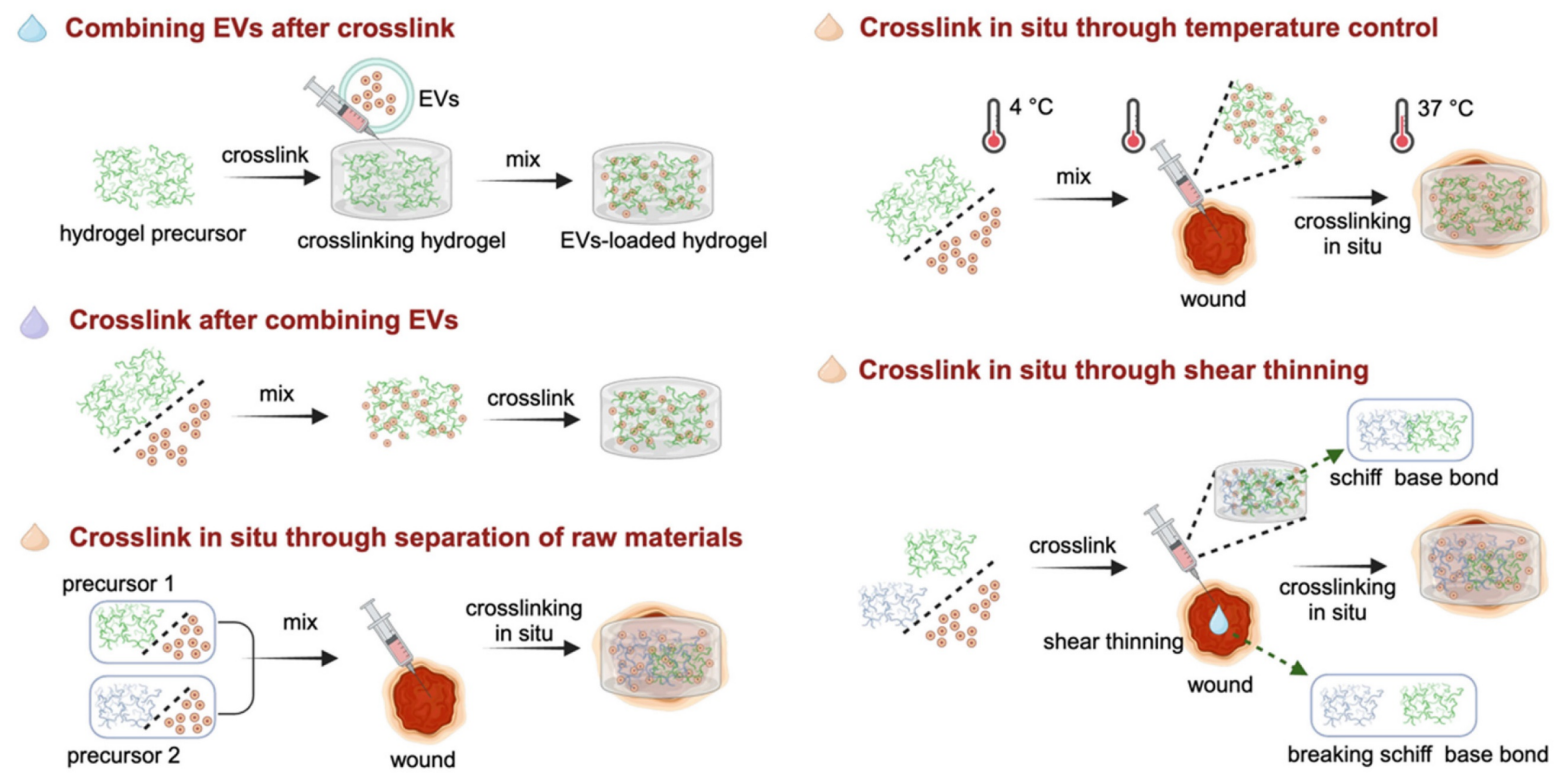
Schematic diagram illustrating the synthesis strategies of EVs combined with hydrogels for accelerating wound healing.

The efficacy of binding techniques is significantly influenced by the method through which hydrogels are crosslinked to generate scaffolds and the timing of the integration of EVs with precursor materials or fully developed hydrogels 100. In **Figure 4** summarizes the synthesis strategies of EVs in combination with hydrogels to accelerate wound healing.

### Combining EVs after crosslink

This technique, known as the “breathing” method, involves the initial crosslinking of raw materials for hydrogels, followed by the introduction of EVs 101. To be specific, this is achieved by dehydrating the hydrogels to create pores. And then, these porous hydrogels are immersed in the EVs contained solution. During the subsequent swelling process of the hydrogels, the EVs become integrated or 'composited' within the hydrogel matrix. This approach enables the effective incorporation of EVs within the hydrogel structure, facilitating their utilization and delivery in subsequent applications. For example, Han *et al.* crosslinked raw materials by ultraviolet radiation to prepare an N-acryloyl glycinamide/gelatin methacryloyl (GelMA)/liponite/glycerol hydrogel, and then introduced the periosteum-derived EVs solution into the hydrogel as a dressing for diabetic wounds 102. In addition, platelet-rich plasma (PRP)-derived EVs were absorbed by cross-linked chitosan/silk hydrogels and found that it effectively enhanced collagen deposition and angiogenesis, thus accelerating the healing of diabetic wounds 103.

Although the method is relatively straightforward, it has several drawbacks. A significant hurdle encountered in the field of hydrogels is ensuring that their pore size is adequate for the absorption of EVs; however, overlarge pore size leads to the rapid release of EVs, resulting in a loss of sustained release capacity 104. Consequently, careful pre-design of composite hydrogels is crucial to ensure optimal release results.

### Crosslink after combining EVs

In this strategy, the EVs solutions are first mixed with the hydrogel precursors, and subsequently crosslinked with the crosslinking agents or without agents. This strategy ensures minimal loss of EVs during the preparation of composite hydrogel dressings, while simultaneously providing precise control over the total amount and proportion of EVs and hydrogels. Additionally, it provides the flexibility to prepare smaller apertures, helping to increase the total amount of EV packaging 100. For crosslinking by agents, the addition of transglutaminase (TGase) as a crosslinker to the EVs and type III collagen solution resulted in crosslinking of the mixture. TGase effectively enhanced the adhesion of EVs to the surface of collagen, ultimately extending the release profiles of EVs within the collagen hydrogel 105. The crosslinking of hydrogel precursors and EVs without agents involves adjusting certain physical conditions, such as temperature and pH value, to promote crosslinking 99, 106.

As the hydrogels swell and degrade, controlled release profiles of EVs occurs. However, certain crosslinking methods, such as exposure to ultraviolet radiation or the application of specific crosslinking agents, may have side effects on the bioactivity of EVs. Therefore, in-depth consideration is required when determining the crosslinking conditions to ensure optimal EVs release and function.

### Crosslink *in situ*

This strategy is similar to the method of “crosslinking after combining EVs”. However, in this particular approach, hydrogels need to possess distinctive attributes such as injectability or thermosensitivity, enabling them to undergo in-situ crosslinking and gelation. This transformation allows them to conformably adapt to the contours of the wounds, ensuring optimal coverage and filling. This approach allows the hydrogel to conform seamlessly to the unique shape and contours of the wound, providing an optimal healing environment. In this section, crosslinking *in situ* is achieved through separation of raw materials, temperature control, and shear thinning.

#### Crosslink *in situ* through separation of raw materials

In this way, *in situ* crosslinking is carried out by first mixing two or more hydrogel precursor materials with EVs individually. This mixing ensures that the EVs are uniformly distributed within the hydrogel precursors. Subsequently, the mixed solutions are simultaneously injected into the wound site, where crosslinking and gelation processes commence spontaneously on the wound surface. For instance, by combining two precursor materials, hydrazide-grafted hyaluronic acid (HAh) and aldehyde-grafted HA (HAa), with the EVs solution, the formation of composite hydrogel was observed followed by *in situ* injection through a dual-chamber syringe, owing to the Schiff base reaction between the hydrazides of HAh and the aldehydes of HAaq 107. This novel *in situ* injectable hydrogel was introduced to greatly improve the diabetic wound healing.

#### Crosslink *in situ* through temperature control

To facilitate *in situ* cross-linking via temperature regulation, a mixture of temperature-responsive hydrogel precursors and EVs is applied directly onto the wound surface. This method offers significant advantages compared to traditional wound treatment methods, as it allows for precise delivery of therapeutic agents directly to the affected area, while also realizing convenient administration and automatic gelation at physiological temperature. Temperature-sensitive hydrogels, such as Pluronic F127, have attracted significant interest in the field of wound healing and tissue regeneration owing to their exceptional characteristics, such as sensitivity to temperature, biodegradability, injectability, and capacity to maintain a moist environment for wounds 108. For example, Zhou *et al*. employed Pluronic F-127 hydrogel to encapsulate ADSCs-EVs for topical administration to a full-thickness cutaneous wound. After fine-tuning the concentration parameters of the precursor solutions, the hydrogel formed in about 17 s at 37 ℃ (the physiological temperature of the wound) 109. This versatile biomaterial construction strategy holds great promise for advancing the development of efficient wound healing therapies.

#### Crosslink *in situ* through shear thinning

In this way, hydrogels should have shear-thinning characteristics to undergo *in situ* crosslinking through shear forces, which provides an alternative method for *in situ* gelling after local injection 110, 111. When no force is applied, the structure of the hydrogel network is stabilized by non-covalent interactions. Owing to the reversible and dynamic interactions, the crosslinked networks were destroyed under shear force, resulting in the liquefaction of the hydrogel. However, the materials can quickly return to their gel state after the external forces are relieved 112, 113. Therefore, these hydrogels prepared in advance *in vitro* undergo solid-liquid transformation under the action of injection force, and then achieve rapid in-situ gelation after injection into the wound. Due to the amino groups in the grafted polyethylene and the aldehydes in the aldehyde pullulan, the Pluronic F127-based hydrogel possessed a shear-thinning ability via hydrogen bonding and Schiff base reactions with the wounds 114. Hence, the combination of EVs with this composite hydrogel enabled the formulation to be easily administered through a syringe directly to the wound region, ensuring well fit to the size and shape of the injured area.

## Multifunctional hydrogel-based engineered EVs delivery for complicated wounds

With the use of hydrogels as a sustained drug release system, combined with EVs treatment, most wounds can be successfully healed 115-117. However, when serious conditions such as chronic diabetic wounds and infected wounds occur, the healing process may be delayed or even blocked. At this time, the strategy of delivering EVs via traditional hydrogels may not achieve satisfactory healing results in these cases. Therefore, the design of functionalized hydrogels with specific wound microenvironment regulatory capabilities, such as antioxidant, anti-inflammatory, immunoregulatory and antibacterial effects, for loading modified engineered EVs may be a potential approach to address the challenge of these complicated wounds. Here, taking diabetic wounds, infected wounds, burn wounds, and scar wounds as examples, we describe the ues of multifunctional hydrogel-loaded engineered EVs to promote wound healing, thus paving the way for the design and application of this novel wound dressing.

### Diabetic wounds

Diabetic foot ulcers (DFUs) are a prevalent and significant complication of diabetes, affecting as many as 25% of individuals living with the disease and presenting a high risk of persistent pain, delayed wound healing, amputation, and even early death 118. Diabetic patients have impaired glucose metabolism leading to a high blood glucose state, which blocks all phases of healing. High blood glucose can disrupt a series of biological responses, including inhibiting the migration, proliferation, and differentiation of skin cells at the wound regions as well as the production of pro-healing factors, promoting the sustained secretion of pro-inflammatory cytokines, ROS accumulation, oxidative stress, immune response disorders, and angiogenesis obstruction, thus delaying the wound healing process 119. Moreover, the harsh diabetic microenvironment not only presents obstacles in maintaining adequate activity and function of EVs, but also hinders their effective targeting and sustained release in the context of wound repair. Currently, the focus of diabetic wound treatment is to create an optimal local microenvironment that promotes and supports the healing process 120. Therefore, designing functionalized hydrogels to modulate excessive inflammation, dysregulated metabolic activity, and macrophage polarization in the DFUs microenvironment, and deliver engineered EVs, may be a potential strategy for promoting DFUs healing.

**Figure 5:**
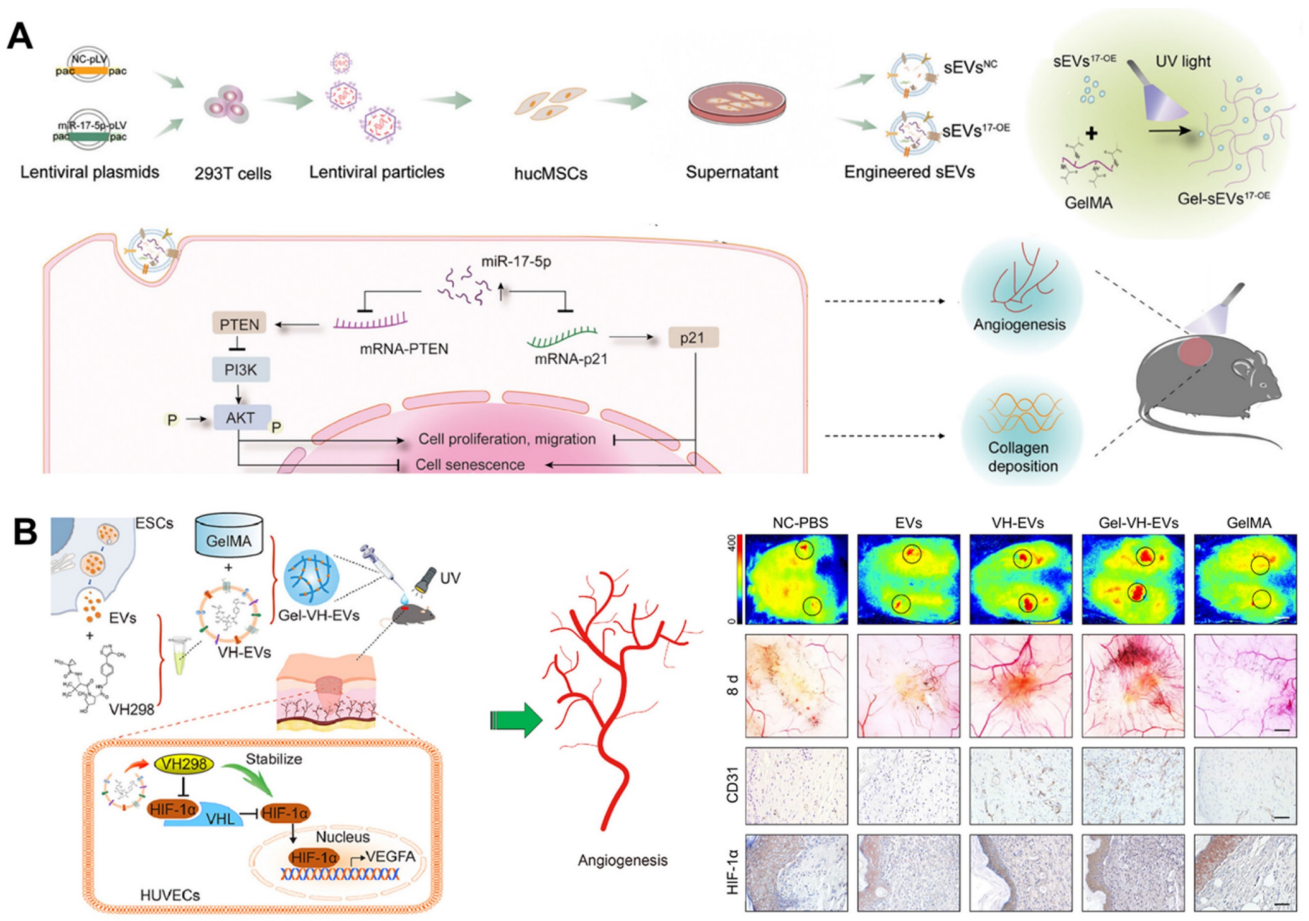
(A) The process of preparing miR-17-5p-engineered EVs loaded GelMA hydrogels and the regulatory mechanisms involved in promoting DFUs healing. Adapted with permission 126. Copyright © 2024, John Wiley & Sons, Inc. (B) VH298-loaded EVs incorporated GelMA hydrogel induced DFUs healing via HIF-1α-mediated angiogenesis. Adapted with permission 128. Copyright © 2022, Elsevier Ltd.

miRNAs, which are carried in EVs, can inhibit the expression of target genes by binding to the 3′ untranslated regions of mRNAs after transcription 121. Studies have indicated that overexpression of miR-17-5p can protect endothelial cell damage induced by high glucose (HG) 122, enhance the angiogenesis of endothelial cells 123, 124, and play a protective role in fibroblasts 125. Therefore, Wei *et al.* fabricated miR-17-5p-engineered EVs and then loaded them in a GelMA hydrogel. This novel bioactive wound dressing improved the biofunctions of HG-induced endothelial cells and fibroblasts by targeting p21 as well as phosphatase and tensin homolog (PTEN) *in vitro*, and effectively promoted DFUs healing by accelerating collagen deposition and blood vessel formation *in vivo* (**Figure 5A**) 126. In addition, VH298 is a small-molecule compound reported by Ciulli *et al.* in 2016, which can serve as a stabilizer of HIF-1α 127. The integration of VH298-loaded EVs into a porous GelMA hydrogel has the potential to significantly extend the retention time up to 15 days, thus promoting the healing of DFUs through HIF-1α-mediated angiogenesis (**Figure 5B**) 128. Therefore, these GelMA hydrogels encapsulating engineered EVs as novel bioactive wound dressings offer an option for DFUs management.

**Figure 6:**
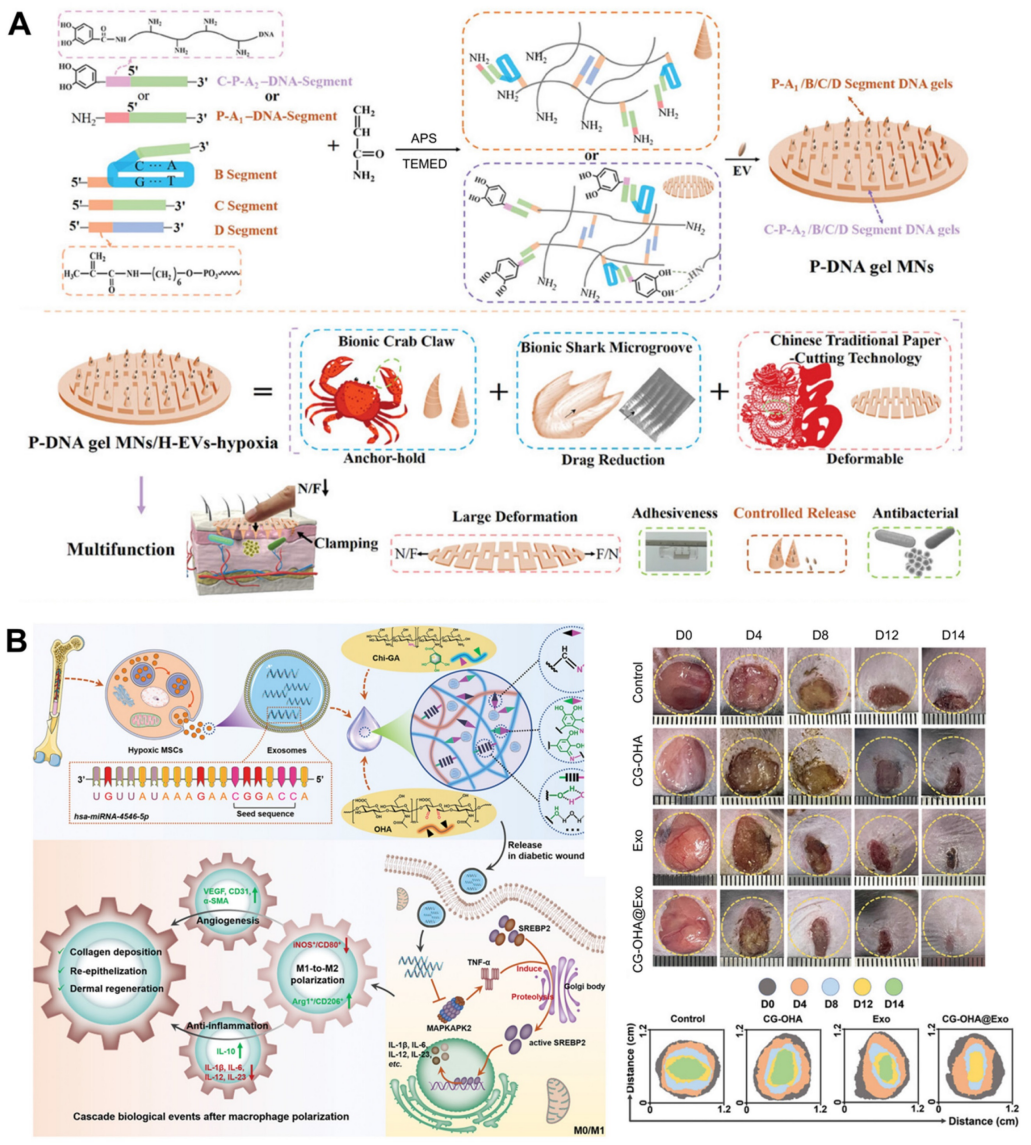
(A) Synthesis process and multifunctional properties of P-DNA gel MNs to incorporate EVs extracted under hypoxia for DFUs. Adapted with permission 131. Copyright © 2023, John Wiley & Sons, Inc. (B) Hypoxic MSCs-derived EVs loaded multifunctional hydrogel accelerated DFUs healing by relieving macrophage dysfunction. Adapted with permission 133. Copyright © 2024, John Wiley & Sons, Inc.

Unlike traditional polymers, deoxyribonucleic acid (DNA) stands out as a natural biopolymer material with exceptional precision in terms of customization. Its unique ability to precisely control the number and order of its units offers a powerful toolbox for creating materials with tailored properties and functionalities, holding great promise for future applications in diverse fields 129, 130. To address the complicated pathological issues of diabetic wounds, e.g., failure to up-regulate pro-healing factors, formation of biological barriers, microangiopathy and cutaneous neuropathy caused by hyperglycemia and hypoxia environment, Zhou *et al.* prepared polypeptide DNA hydrogel microneedles (P-DNA gel MNs) to incorporated EVs extracted under hypoxia. This multifunctional hydrogel-loaded engineered EVs strategy can activate immune regulation, promote neurogenesis and angiogenesis, and accelerate DFUs healing with high quality by alleviating the wound microenvironment, scavenging free radicals, and alleviating inflammation (**Figure 6A**) 131. Compared with normoxia, the survival and proliferation of MSCs were significantly enhanced after hypoxia induction, and hypoxia-induced MSCs-derived EVs can inhibit inflammation and promote DFUs healing through the PI3K/AKT signaling pathway 132. A multifunctional hydrogel with antibacterial and antioxidant abilities consisting of gallic acid (GA)-conjugated chitosan (Chi-GA) and partially oxidized hyaluronic acid (OHA) was designed as a vehicle for hypoxic BMSCs-derived EVs. The composite dressing relieved macrophage dysfunction during DFUs healing by inducing polarization toward M2 phenotype, possibly because the exosomal miR-4645-5p and the antioxidant ability of the hydrogel synergistically restrained SREBP2 activity in the macrophages (**Figure 6B**) 133.

In general, the use of miRNA-engineered EVs or hypoxia-pretreated MSCs-derived EVs combined with multifunctional hydrogels as bioactive dressings is an alternative strategy for improving wound healing and provides a reference for the basic mechanism of clinical transformation in managing DFUs.

### Infected wounds

Wound infection poses a significant risk within the healthcare system. Opportunistic pathogens have the ability to invade, colonize, and proliferate in the wound region, leading to potential infection in a variety of wound types, such as burns and traumas 134-136 Bacterial infections can cause dramatic changes in the microenvironment around the wound, including increased levels of bacterial secretory enzymes and decreased microenvironment pH due to acidic metabolites 135. Infected wounds can lead to a protracted healing process and, in severe cases, may result in complications such as magnified inflammation, septicemia, osteomyelitis, disability, and even death 137, 138. Traditional pathways and the overuse of antibiotics have contributed to the increase in antibiotic resistance. The emergence of multidrug-resistant conditions is predicted to pose great challenges. Consequently, there is an urgent need to devise innovative drug delivery systems capable of regulating drug release within the target, ultimately mitigating antibiotic resistance.

Multifunctional hydrogels with inherent antimicrobial activity or antimicrobial delivery are considered as alternative strategies to overcome this address 139. When using EVs to manage infected wounds, the development of smart hydrogel dressings that can achieve on-demand antibacterial properties has broad application prospects in the future.

**Figure 7:**
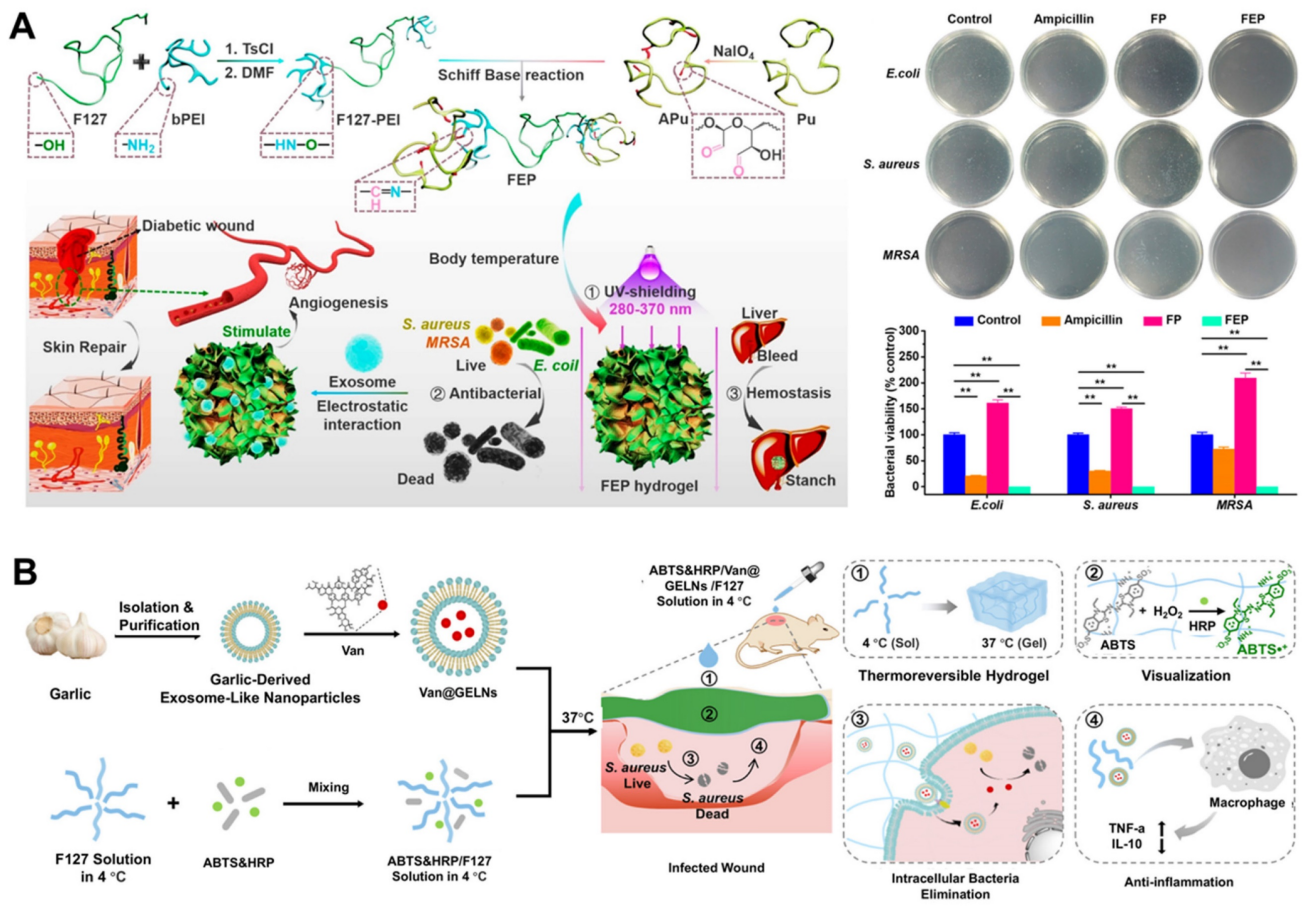
(A) Illustration of the synthesis process of polysaccharide-based multifunctional hydrogels and nanoscale EVs-loaded wound dressings and their potential application in promoting infected wound healing. Adapted with permission 114. Copyright © 2019, ACS Publications. (B) Scheme of the HRP&ABTS/Van@GELNs/F127 hydrogel for *S. aureus* infection visualization and management of the wound. Adapted with permission 142. Copyright © 2024, ACS Publications.

Driven by the great clinical need, Wang *et al.* designed a polysaccharide-based multifunctional hydrogel with thermosensitivity, injectability, self-healing properties, and adhesion to incorporate MSCs-derived nanoscale EVs through a reversible Schiff base reaction of electrostatic interactions. The composite dressing possessed sufficient antibacterial ability for multidrug-resistant bacteria, hemostatic capacity, excellent UV-shielding property, and pH-responsive EVs release profiles, thus inducing wound healing (**Figure 7A**) 114. Garlic-derived exosome-like nanovesicles (GELNs) not only have various biofunctions, such as anti-inflammatory and anti-antibacterial effects, but also have an efficient capacity for cellular internalization as potential nanocarriers to deliver specific cargoes 140, 141. Four methods (including freeze-thaw, sonication, electroporation, and incubation) were used for embedding vancomycin into GELNs to prepare engineered EVs (Van@EVs), which were then encapsulated them in a Pluronic F127-based thermosensitive and visible hydrogel dressing. This multifunctional hydrogel loaded with Van@EVs enabled efficient healing and direct visualization of *S. aureus* infected wounds (**Figure 7B**) 142.

**Figure 8:**
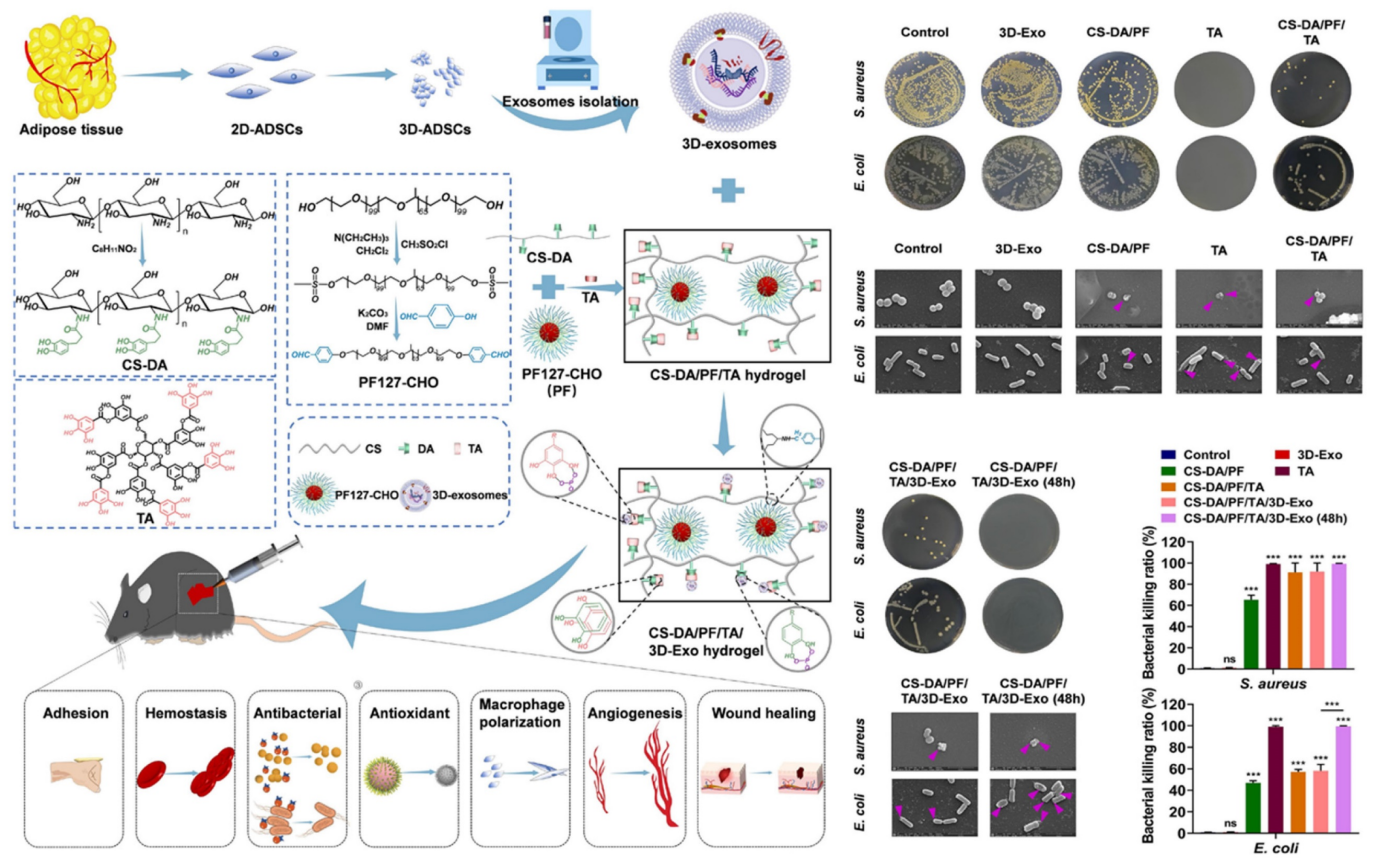
Illustration of the preparation of CS-DA/PF/TA/3D MSCs-EVs hydrogel and its excellent antibacterial ability for wound treatment. Adapted with permission 144. Copyright © 2023, Elsevier Ltd.

To obtain higher quality EVs, 3D cell culture can achieve greater yields of EVs and better healing results than 2D cell culture 143. Chitosan-grafted-dihydrocaffeic acid (CS-DA) and benzaldehyde-terminated Pluronic F127 (PF127-CHO) were combined by dynamic Schiff base bonding, and then fused tannic acid (TA) and 3D cultured MSCs-derived EVs. As a result, this composite dressing exerted various performance, such as antibacterial, tissue adhesive, hemostatic, anti-inflammatory, and antioxidant effects, to promote neovascularization and wound healing (**Figure 8**) 144. TA has a large number of phenolic hydroxyl groups, which have been shown to hinder the synthesis of bacterial cell walls and disrupt the membrane structures. This disruption leads to altered permeability and results in the impairment of barrier function 145. Furthermore, TA has the ability to suppress extracellular microbial enzymes, thus depriving microorganisms of essential substrates for growth and ultimately interrupting microbial metabolism by inhibiting oxidative phosphorylation, all of which contribute to its excellent antibacterial properties 146. Due to the excellent antibacterial property of TA, the CS-DA/PF/TA/3D MSCs-EVs hydrogels show great potential in the management of infected wounds.

In conclusion, endowing a composite system with superior antibacterial properties, whether through modification of EVs or optimization of hydrogel formulations, is a critical strategy for addressing the issue of infectious wound healing.

### Other complicated wounds

In addition to diabetic wounds and infected wounds, multifunctional hydrogel-based engineered EVs delivery can also be applied to some other complicated wounds, such as burn wounds and scar wounds. ADSCs-EVs have shown great potential in regenerative medicine and have been shown to benefit wound repair, such as burns 147. Zhu *et al.* designed a high-performance ADSCs-EVs sustained release hydrogel dressing for burn wound healing by loading 3D-printed microfiber culture-derived EVs in a highly biocompatible hyaluronic acid. Compared with conventional 2D plate culture (2D-EVs) and microcarrier culture (2.5D-EVs), 3D-printed microfiber culture promoted keratinocytes and human umbilical vein endothelial cells (HUVECs) proliferation and migration, as well as induced angiogenesis of HUVECs. Additionally, hydrogel-loaded 3D-EVs promoted burn wound healing to a greater extent than did 2D-Exos or 2.5D-Exos, enhancing the burn wound healing rate and inducing collagen remodeling 148. To better control the infection and scarless healing in the burn wounds, as well as long-term preservation and activity maintenance of EVs, Yang *et al.* proposed the utilization of rapid freeze-dry-thaw macroporous hydrogels for the encapsulation of MSCs-EVs combined with an antimicrobial peptide coating. In the deep second-degree burn infection models, this composite dressing could effectively regulate the behaviors of various skin-related cells, thus inducing tissue repair and inhibiting scar formation 149.

For scarless skin healing, MSCs-EVs incorporated with biofunctional hydrogels exert immunomodulatory effects by driving macrophages toward an anti-inflammatory and anti-fibrotic (M2c) phenotype 150. At present, the application of multifunctional hydrogel-based engineered EVs delivery is mainly focused on diabetic wounds and infected wounds, and there are few reports on the use of these materials to burn wounds and scar wounds. However, we believe that with the advancement of synthesis technology of multifunctional hydrogels and the development of engineered EVs preparation, this multifunctional hydrogel-based engineered EVs delivery strategy will have an increasing number of applications in burn wounds and scarless healing.

## Summary and Perspectives

As discussed in this review, the strategy of multifunctional hydrogel-based engineered EVs delivery has been well demonstrated to promote the repair of complicated wounds, including diabetic wounds, infected wounds, burn wounds, and scar wounds. Faced with these wounds, functionalized hydrogels with specific wound microenvironment regulatory capabilities, such as antimicrobial, anti-inflammatory, and immune regulation, used for loading engineered EVs, provide potential approaches for addressing these healing challenges. However, developing EVs-loaded hydrogel dressings still poses some potential obstacles: (1) EVs: standardized protocols for producing, extracting, modifying, and storing EVs need to ensure the stability and reliability of therapeutic EVs; (2) hydrogels: developing hydrogel formulations with better properties, such as optimized biocompatibility and release performance; and (3) synthesis strategies: improving preparation methods to decrease adverse effects on EVs and further optimize the properties of composite dressings to adapt to more personalized and customized clinical applications.

Apart from ensuring consistent yield and batch quality, the process of producing and extracting EVs is still in the experimental stages and involves limited purification. The techniques of ultracentrifugation and differential centrifugation, which can achieve clinically acceptable purity, are hindered by high costs, lengthy processing times, and low yields 151. Nevertheless, as technology continues to advance and equipment continues to evolve, it is anticipated that high-precision, high-throughput purification technologies such as microfluidics and immunomagnetic bead techniques will emerge as the industry standards for EVs production and extraction. This shift could revolutionize the purification process for EVs in terms of efficiency and effectiveness 95. The establishment of standardized processes is imperative for the utilization of EVs in wound treatment. When employed as a therapeutic delivery vehicle, different cultivation, extraction, and engineering modification procedures can impact EVs viability and introduce foreign contaminants into the products. Additionally, variations in characterization and counting strategies can affect the quality control of EVs 152. Moreover, at present, composite hydrogels are usually used immediately after preparation, and related storage methods are lacking. Despite recent advancements in EVs research, there is still a deficiency in comprehensive understanding of EV functions and mechanisms, as well as the composition and characterization of EVs subgroups. Therefore, developing standardized protocols for the sourcing, collection, processing, extraction, characterization, and analysis of EVs is essential for mass production and clinical translational applications 153.

**Figure 9:**
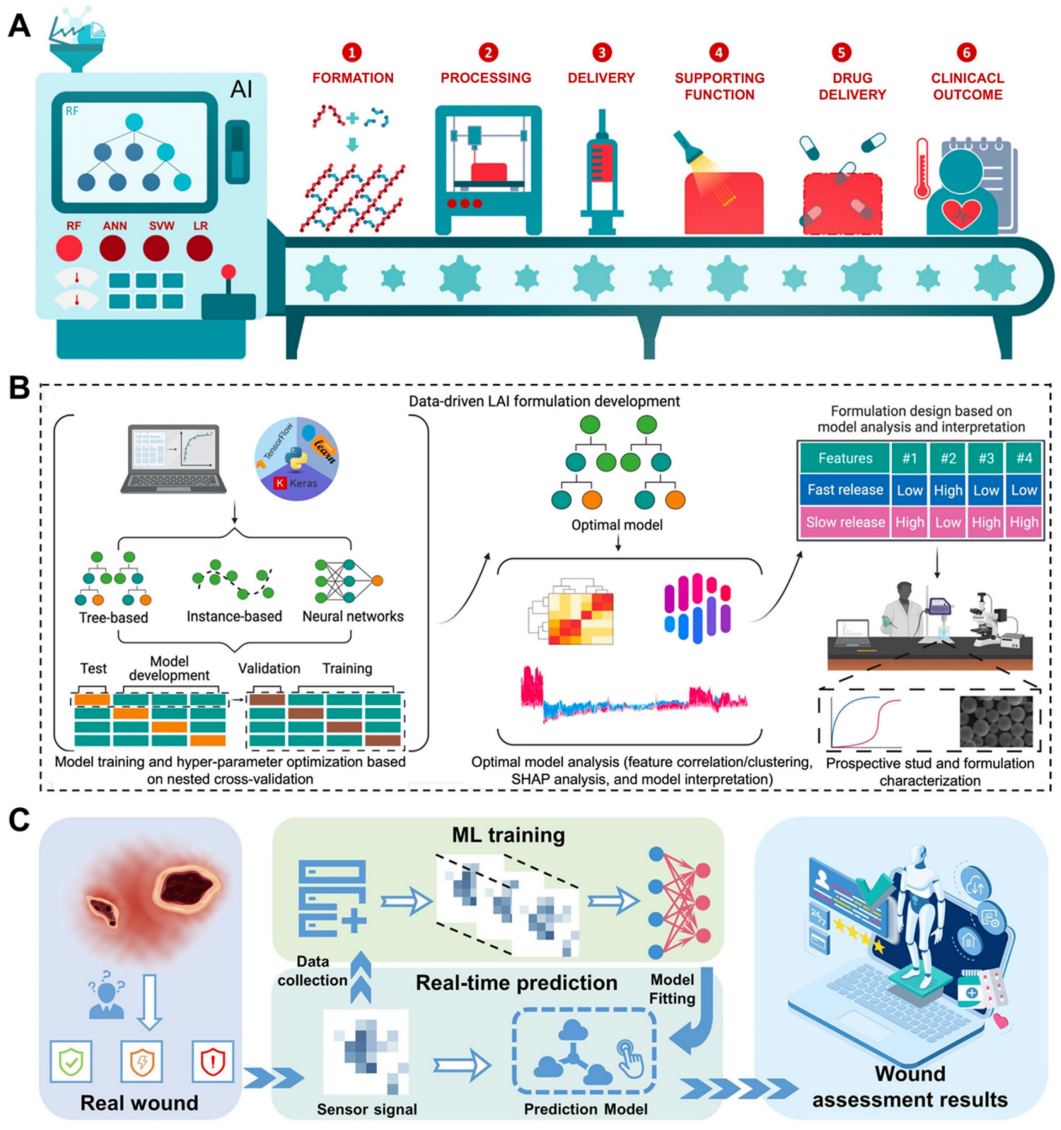
(A) Through the application of AI strategies, the preparation of hydrogels as drug delivery systems has been improved in multiple stages. Adapted with permission 157. Copyright © 2022, Elsevier Ltd. (B) Training and analysis of AI models to accelerate the manufacturing cycle of novel long-acting injectable systems. Adapted with permission 158. Creative Commons CC BY license. (C) Schematic illustration of intelligent wound monitoring by multifunctional hydrogel dressings, such as wound recognition, real-time status supervising, and customized wound management. Adapted with permission 159. Copyright © 2022, Elsevier Ltd.

Another challenge that needs to be highlighted is how to explore hydrogel formulations with improved properties to better adapt to the complicated wound microenvironment and thus achieve better healing results. Artificial intelligence (AI) has the potential to revolutionize the design and preparation of hydrogels 154. In terms of advantages, the AI approach can efficiently predict and optimize the composition and properties of hydrogels. Through the use of AI models, parameters can be automatically adjusted during the hydrogel preparation process to obtain the optimal formulations. This capability holds promise for streamlining the overall process and improving the quality and efficiency of hydrogel production 155, 156. Specifically, the potential for AI to revolutionize the workflow of hydrogels as drug delivery vehicles has been highlighted by developing predictive models, algorithm optimization, and image processing and recognition. The use of AI has shown promise in accurately predicting hydrogel formation, optimizing hydrogel performance, and fine-tuning drug release profiles (**Figure 9A**) 157. The AI algorithm predictive models can guide the preparation of novel long-acting injectable formulations effectively. Using this data-driven approach holds the potential to reduce time and costs associated with drug formulation exploitation (**Figure 9B**) 158. These advancements represent a significant step forward in the application of AI within the field of hydrogel-based drug delivery systems. Furthermore, AI has the capacity to substantially influence the utilization of composite hydrogel dressings. The image processing and recognition abilities of AI can automatically assess and diagnose the wound or lesion region, ultimately aiding in the identification of appropriate hydrogel dressings and preparation protocols (**Figure 9C**) 159. In addition, AI has the potential to play a crucial role in enhancing the performance of hydrogels and their application in various environments. Through the use of sensor networks and data acquisition systems, it has become possible to monitor key parameters such as temperature, humidity, and pH value in real time. By integrating AI algorithms into this monitoring process, any deviations from the expected conditions can be swiftly identified, allowing timely interventions to safeguard the characteristics and stability of the hydrogels 160-163. This approach has the potential to revolutionize the development and application of hydrogels in biomedical settings. In addition to AI-energized hydrogel manufacturing and optimization strategies, 3D printed multifunctional hydrogel-based engineered EVs systems are also considered suitable for more personalized and customized clinical applications.

In the area of wound healing, 3D bio-printing provides a precise method for creating custom-shaped hydrogel materials that are specifically designed to match the contours of the wound. The versatility of 3D bio-printing method offers a potential strategy for optimizing the preparation process and properties of composite hydrogel dressings, facilitating controlled EVs delivery to accelerate wound healing efficiently 164-166.

In summary, the use of multifunctional hydrogels loaded with engineered EVs is a promising approach for promoting wound healing. Moving forward, a more comprehensive understanding of the properties of EVs and hydrogels will greatly improve the efficacy of complicated wound healing. Furthermore, as technology continues to advance, the combination of AI-energized material design and high-precision 3D bio-printing technology will significantly improve the effectiveness of EVs-loaded hydrogels and expand their potential clinical use in complicated wounds. This advancement is anticipated to open up new possibilities for treating tissue damage and promoting regenerative medicine.

## Figures and Tables

**Scheme 1:**
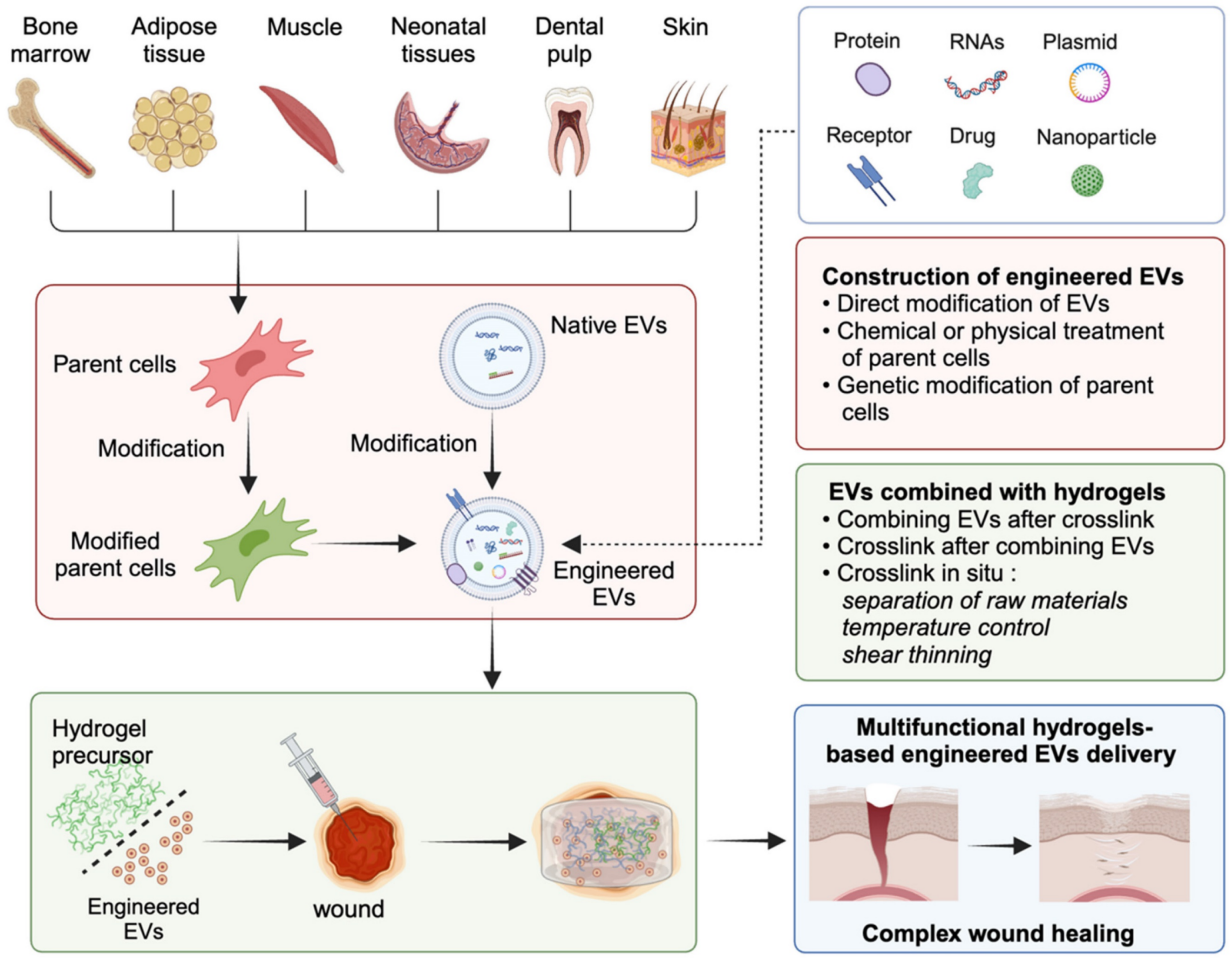
Schematic diagram of multifunctional hydrogel-based engineered EVs delivery for enhancing complicated wound healing.
